# 3D-Printed PEEK/Silicon Nitride Scaffolds with a Triply Periodic Minimal Surface Structure for Spinal Fusion Implants

**DOI:** 10.1021/acsabm.3c00383

**Published:** 2023-08-10

**Authors:** Xiaoyu Du, Sean Ronayne, Seunghun S. Lee, Jackson Hendry, Douglas Hoxworth, Ryan Bock, Stephen J. Ferguson

**Affiliations:** † Institute for Biomechanics,ETH Zurich, Zurich 8093, Switzerland; ‡ SINTX Technologies, Inc., Salt Lake City, Utah 84119, United States

**Keywords:** silicon nitride, PEEK, 3D printing, implants, spine

## Abstract

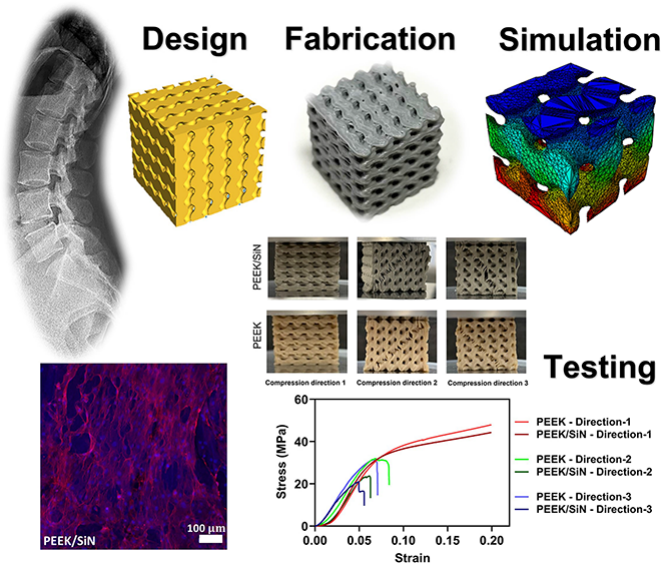

The issue of spine-related disorders is a global healthcare concern that requires effective solutions to restore normal spine functioning. Spinal fusion implants have become a standard approach for this purpose, making it crucial to develop biomaterials and structures that possess high osteogenic capacities and exhibit mechanical properties and dynamic responses similar to those of the host bone. This study focused on the fabrication of 3D-printed polyether ether ketone/silicon nitride (PEEK/SiN) scaffolds with a triply periodic minimal surface (TPMS) structure, which offers several advantages, such as a large surface area and uniform stress distribution under load. The mechanical properties and dynamic response of PEEK/SiN scaffolds with varying porosities were evaluated through mechanical testing and finite element analysis. The scaffold with 30% porosity exhibited a compressive strength (34.56 ± 1.91 MPa) and elastic modulus (734 ± 64 MPa) similar to those of trabecular bone. In addition, the scaffold demonstrated favorable damping properties. The biological data revealed that incorporating silicon nitride into the PEEK scaffold stimulated osteogenic differentiation. In light of these findings, it can be inferred that PEEK/SiN TPMS scaffolds exhibit significant potential for use in bone tissue engineering and represent a promising option as candidates for spinal fusion implants.

## 1 Introduction

The human spine serves a crucial role in supporting the upper body by transmitting compressive and shear forces to the lower body during daily activities.^1^ However, as individuals age, various spine-related disorders commonly arise, such as intervertebral disc degeneration, disc herniation, spinal stenosis, and facet arthritis, which are recognized as significant factors contributing to the development of low back pain.^2^ Chronic low back pain afflicts numerous individuals, impeding their daily activities and reducing their overall quality of life, thus creating a global healthcare concern.^3^ To alleviate pain and stabilize degenerated segments, spinal fusion surgery is considered the gold-standard treatment. This procedure typically involves the implantation of an intervertebral fusion cage, which offers direct axial load support, preserves intervertebral and foraminal space, and ultimately promotes osseointegration, ideally through an osteoconductive or osteogenic effect, between adjacent vertebrae.^4,5^

Silicon nitride has several compelling properties, such as high strength, osteoconductivity, and antibacterial effects, which are essential for the development of spinal implants.^6−8^ A 30-year clinical study demonstrated that silicon nitride is a biocompatible material that is capable of integrating with bone tissue and promoting osteogenic activity.^9,10^ In addition, Lee et al.^11^ evaluated and compared the biological response of commonly used biomaterials for spinal implants *in vitro*: silicon nitride and surface-textured silicon nitride, zirconia toughened alumina, titanium alloy Ti6Al4 V, and polyether ether ketone (PEEK). In comparison to the other groups, silicon nitride exhibited a greater osteogenic response and reduced levels of inflammation. Overall, extensive clinical results have shown that silicon nitride is effective as a spinal implant.^6,12,13^

However, the disadvantage of using dense silicon nitride as a spinal implant is that the high elastic modulus of silicon nitride may lead to stress shielding, which can result in implant subsidence or bone atrophy. To address this issue, porous silicon nitride was fabricated to reduce stress shielding and facilitate osseointegration. In our previous work,^14^ we investigated the influence of porosity on the mechanical properties of silicon nitride and found that porous silicon nitride scaffolds may be a promising approach to reduce stress shielding in comparison to bulk silicon nitride or conventional metal implants. However, even with 70% porosity, the elastic modulus of porous silicon nitride remained significantly higher than that of human cancellous bone.

Additionally, biomechanical studies have highlighted the importance of damping in optimizing implant performance in the spinal region.^15−17^ Given the unique dynamic properties of the spinal system, the damping characteristics of spinal implants are of particular significance.^18^ The dynamic properties of dense and porous silicon nitride were evaluated in our previous study,^14^ and both showed low damping properties. Specifically, their energy dissipation capacity was significantly inferior to that of natural spinal tissue. Despite the fact that the silicon nitride bioceramic used in the study had a porosity of approximately 70%, there was a high proportion of closed pores formed by elongated grains, which may have adversely affected its energy dissipation capacity. Therefore, further efforts are needed to improve the damping properties of silicon nitride for enhanced spinal implant performance.

Cellular structures such as periodic cells and stochastic foams are widely used as energy absorbing structures.^19^ Recently, the triply periodic minimum surface (TPMS) structure has attracted the attention of many researchers. It is a class of mathematically defined surfaces with periodicity in *X*, *Y*, and *Z* directions and an average curvature of 0 at every point on the surface. The surface is partitioned into two infinitely intertwined domains, while the whole structure remains an open cavity.^20^ The TPMS structure possesses several notable advantages, including a large surface area, a favorable strength-to-weight ratio, a uniform stress distribution under load-bearing conditions, and a high energy absorption capacity with low relative density.^21,22^

PEEK is one of the main materials clinically used for fusion cages, and it is a polymer that is biomechanically similar to cortical bone, offering advantages in terms of load distribution.^23,24^ However, PEEK does not bind directly to bone due to its chemical inertness and hydrophobicity.^25^ Bulk incorporation of osteoconductive materials into the PEEK matrix is a potential strategy to mitigate the formation of fibrous tissue between PEEK and bone.^26^ In addition, PEEK has good ductility and toughness. Composites of PEEK and silicon nitride can overcome the brittleness of silicon nitride and may have better energy dissipation capabilities.

Here, we propose using silicon nitride and PEEK to mimic native trabecular bone. PEEK filaments containing 10 wt % silicon nitride were produced. With the development of advanced additive manufacturing technology, PEEK/SiN scaffolds with a triply periodic minimal surface structure (namely, PEEK/SiN TPMS scaffold) could be 3D-printed. Finally, their mechanical and biological properties were evaluated.

## 2 Materials and Methods

### 2.1 Materials

PEEK filaments with a diameter of 1.75 mm were purchased from Henan Suwei Electronic Technology Co., Ltd. (Zhengzhou, China). PEEK/SiN material is produced by compounding an extremely fine particulate form of silicon nitride bioceramic (10 wt %) into an implant grade PEEK matrix. The filament with a diameter of 1.75 ± 0.05 mm was fabricated by Ensinger Inc. (Nufringen, Germany), where the silicon nitride powder was a sintered, β-phase material with a median particle size of approximately 0.8 μm supplied by SINTX Technologies Inc. (Salt Lake City, USA).

### 2.2 Design of TPMS Structures

The TPMS structure can be approximated mathematically. In this study, we used a gyroid lattice whose surface is described by the following approximation:
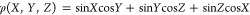
1where *X* = 2απ*x*, *Y* = 2βπ*y*, and *Z* = 2γπ*z*. The unit cell size in the *x*, *y*, and *z* directions of the structure is controlled by parameters α, β, and γ. The function φ(*X*, *Y*, *Z*) is an isosurface evaluated at an isovalue that controls the cell relative density.

In this work, an open-source software MSlattice (https://www.oraibkhitan.com/) was used to generate the TPMS gyroid model. The unit cells of each TPMS gyroid solid structure are cubes with a side length of 3 mm, taking into account the printing resolution of the FDM machine. Specimens for mechanical testing consisted of an array of 5 × 5 × 5 unit cells, producing a lattice of 15 × 15 × 15 mm. For cell tests, the samples were disc-shaped with a diameter of 15 mm and a height of 3 mm.

### 2.3 Fabrication of PEEK/SiN and PEEK TPMS Scaffolds by 3D Printing

TPMS scaffolds were fabricated by fused deposition modeling (FDM), a material extrusion process that employs G-codes to guide the movement of a heated nozzle, which pushes the fused filament through to construct the scaffold in a layer-by-layer fashion. The nozzle temperature for printing PEEK and PEEK/SiN scaffolds was set at 440 and 415 °C, respectively. The PEEK/SiN filament requires a printing temperature lower than that of the pure PEEK filament. The reason for this could be that the thermal conductivity of silicon nitride is higher, resulting in a lower viscosity. STL files were imported into a slicing software (CreatWare V6.5.2), and G-Codes were generated. The detailed printing parameters are summarized in Table 1. Finally, the G-Code file was sent to a CreatBot F430 3D printer (Henan Suwei Electronic Technology Co., Ltd.) for printout. Prior to printing, a nanopolymer adhesive (Visionminer, USA) was applied to the build surface in order to eliminate lift and warpage of the PEEK-based materials. All TPMS scaffolds are self-supporting, so there is no need for a support structure during the printing process.

**Table 1:** Printing Parameters

printing parameters	PEEK	PEEK/SiN
layer height (mm)	0.2	0.2
extrusion width (mm)	0.4	0.4
fill density (%)	100	100
print speed (mm/s)	30	30
nozzle temperature (°C)	440	415
bed temperature (°C)	140	140
chamber temperature (°C)	70	70

### 2.4 Characterization of PEEK/SiN and PEEK TPMS Scaffolds

The morphology and microstructure of the PEEK/SiN and PEEK scaffolds were examined by using a scanning electron microscope (SEM, FEI Quanta 600 FEG, USA). Before the examination, each sample underwent coating with Au/Pd (80/20) through a sputter coater (Leica EM ACE600 Sputter Coater, Germany). Moreover, to investigate the internal structure of the TPMS scaffolds, microcomputed tomography (micro-CT 100, Scanco Medical, Br̈ttisellen, Switzerland) was performed with a voxel size of 17.2 μm, 70 kVp, 114 μm, and 8 W. Porosity (*P*) of the printed porous TPMS scaffolds was calculated using the following equation:
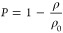
2where ρ is the apparent density of the porous TPMS scaffold and ρ_0_ is the bulk density of the dense TPMS scaffold (nonporous). The determined porosities were then compared to the theoretical porosities of the design models.

### 2.5 Quasi-Static Mechanical Tests

TPMS scaffolds with 30, 50, and 70% porosity were tested in vertical compression to a displacement of 5 mm using a material testing machine with a load cell of 30 kN (Instron 5567, USA) at a speed of 1 mm/min. Quasi-static compression tests were conducted using an unconfined setup between two parallel smooth plates while recording force and displacement signals. The load–displacement curves obtained were subsequently converted into stress–strain curves based on the original dimensions of the scaffold.

In addition, to investigate the mechanical properties of the printed scaffolds in different compression directions, PEEK and PEEK/SiN scaffolds with 30% porosity were selected for testing since they showed better mechanical properties than the other two groups. The samples were observed under a microscope (Olympus SZX 9, Japan) from different views and compressed from 3 different directions (Figure 4). Compression direction 1 was parallel to the build direction, while compression directions 2 and 3 were perpendicular to the build direction. The compression tests were performed under displacement control at a speed of 1 mm/min.

Additionally, the interfacial strength between the printed layers was evaluated by means of a custom-made interfacial shear setup, as depicted in Figure 4A, which was integrated with an Instron testing machine. The tests were conducted at a constant speed of 1 mm/min. The interfacial shear strength was computed by dividing the fracture load at failure by the contacted compression area.

### 2.6 Progressive Loading Tests

PEEK/SiN TPMS scaffolds with 30 and 50% porosity and dimensions of 15 × 15 × 15 mm were subjected to progressive loading tests. The experiments were performed on a dynamic materials testing machine (Instron E10000, 10 kN load cell, Instron, UK) in air and at room temperature. A progressive loading scheme was employed, with the specimens subjected to a maximum displacement of 1.5 mm in increasing steps of 0.3 mm per step, followed by full unloading between loading steps. The displacement rate was kept constant at 1 mm/min. Subsequently, stress–strain values were calculated.

### 2.7 Finite Element Analysis (FEA)

The mechanical properties of the TPMS scaffold were evaluated by FEA under compressive loading conditions. A displacement equivalent to 5% strain was applied to the top surface of the scaffold with the bottom surface fully constrained. A unit cell of the gyroid structure and a cube with a 2 × 2 × 2 cell array were used for the FEA. The material (PEEK/SiN) was assumed to be isotropic and linearly elastic with a Poisson ratio of 0.39, a density of 1.25 g/cm^3^, and an elastic modulus of 1860 MPa. The density and elastic modulus were obtained by experimental testing of printed dense cubic samples. Abaqus software (ABAQUS 6.4.1, Hibbit, Karlsson and Sorenson Inc., USA) was used to perform linear static analysis using a mesh with 10 node tetrahedral elements. The mechanical behavior was examined by plotting the Von Misses stresses, while the elastic modulus of the porous scaffold was calculated from the reaction force and displacement data.

### 2.8 Cell Proliferation Test

PEEK/SiN and PEEK scaffolds with a diameter of 15 mm and a height of 3 mm were 3D-printed and sterilized by autoclave. Mouse preosteoblast cells (MC3T3-E1) were obtained from the University of Zurich (Switzerland). Cells (5 ×10^3^) were seeded on the scaffolds initially and cultured with the growth medium (1% antibiotic-antimycotic and 10% fetal bovine serum in the minimum essential medium α without ascorbic acid). For cell proliferation assessment, a PrestoBlue assay kit (ThermoFisher, USA) was used, following the manufacturer’s protocol. After cell attachment, the culture medium was replaced with the assay medium containing a 10% PrestoBlue solution and 90% growth medium. After incubating for 30 min, the medium was collected, and the growth medium was added back. The collected assay medium (100 μL) was analyzed by using fluorescence spectroscopy at an excitation wavelength of 560 nm and an emission wavelength of 590 nm. The same procedure was performed on days 1, 3, and 7.

### 2.9 Actin/DAPI Staining and Alizarin Red S (ARS) Staining

PEEK/SiN and PEEK TPMS scaffolds with 30% porosity were seeded with 2 × 10^4^ MC3T3-E1 cells and cultured with the growth medium for 5 days. Following the standard protocol, actin and nuclei were stained with Alexa Fluor 568 Phalloidin (Thermofisher, USA) and DAPI (Thermofisher, USA), respectively. Subsequently, the scaffolds were imaged using a confocal laser scanning microscope (CLSM) (Zeiss, LSM 780 upright).

For ARS staining, osteogenic medium was prepared by supplementing growth medium with 50 μg/mL l-ascorbic acid, 10 mM glycerol-2-phosphate disodium salt hydrate, and 100 nM of dexamethasone. Subsequently, 2 × 10^4^ MC3T3-E1 cells were seeded onto the scaffolds and cultured with the osteogenic medium, which was refreshed every other day. After 10 days of osteogenic induction, the ARS kit was employed to measure the mineral content following the standard protocol. Finally, the mineral content was measured by eluting ARS with 10% cetylpyridinium chloride, and optical density (OD) was recorded at 562 nm using a microplate reader.

### 2.10 Real-time Quantitative PCR (RT-qPCR) Analysis

The present study also aimed to investigate the expression of osteogenic-related genes, including alkaline phosphatase (ALP), osteocalcin (OCN), collagen type I (COL1), and runt-related transcription factor 2 (RUNX2), using quantitative reverse transcription polymerase chain reaction (RT-qPCR).^27^ MC3T3-E1 (2 × 10^4^) were seeded on the scaffolds and cultured with the osteogenic medium as described in Section 2.8. The total RNA of osteogenically differentiated preosteoblasts at days 7 and 14 was extracted and purified using the RNeasy Plus Mini Kit (Qiagen Inc., USA). The quality and quantity of the RNA were assessed by using a Nanodrop spectrophotometer (ND-1000 UV–vis Spectrophotometer; Nanodrop Technologies). The extracted RNA was then transcribed into cDNA. The expression levels of osteogenic genes were measured using TaqMan gene expression assays. Three groups of parallel experiments were performed and averaged independently. Gene expression was calculated using the following formula: *P* = 2^–(normalized average *Ct*)^ × 100, where the average cycle threshold of each gene was normalized against the *Ct* value of *GAPDH* (glyceraldehyde-3-phosphate dehydrogenase).

### 2.11 Statistical Analysis

All experimental procedures were conducted in triplicate. The data were expressed as mean values with standard deviations. Grouped data, including the mechanical test, PrestoBlue assay, and PCR tests, were analyzed using two-way analysis of variance (2-way ANOVA), followed by Sidak’s multiple comparisons test. The ARS staining results were analyzed by using the Mann–Whitney test. All statistical analyses were performed using GraphPad Prism 8.2.0 software (GraphPad Software Inc., USA). Statistical significance was set at a level of *p* ≤ 0.05.

## 3 Results

### 3.1 Characterization of PEEK/SiN and PEEK TPMS Scaffolds

**Figure 1:**
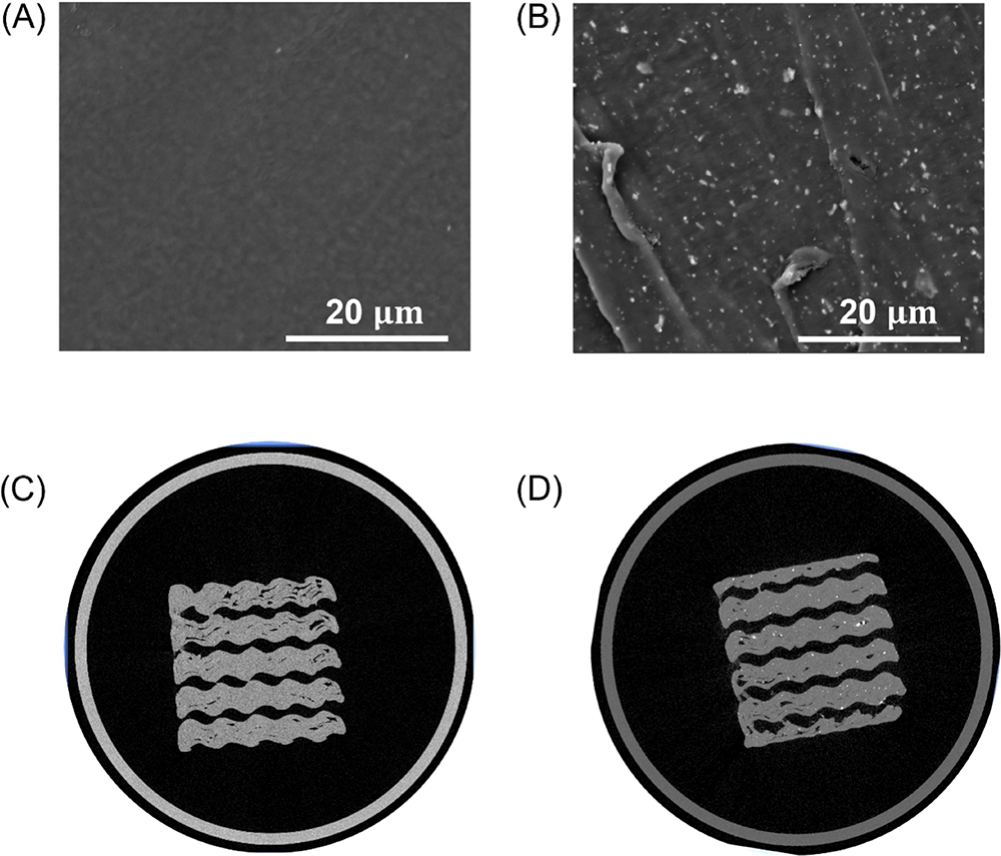
SEM image of (A) PEEK scaffold and (B) PEEK/SiN scaffold; micro-CT image of (C) PEEK scaffold and (D) PEEK/SiN scaffold.

The surface morphologies of the PEEK and PEEK/SiN scaffolds are shown in Figure 1. As can be clearly observed, the silicon nitride powders were successfully embedded in the PEEK matrix. Following the fabrication process, the samples were weighed, and their actual porosity was determined and compared to the porosity of the original designed models. Table 2 shows the deviation in porosity between the designed and the actual printed scaffolds with a slight decrease in porosity for most samples, except for the PEEK/SiN scaffold group with 70% porosity. The observed deviations in the porosity range between approximately −7 and 2%. Of all the groups, the PEEK/SiN TPMS scaffold with 30% porosity had the most similar value of porosity to the designed model. However, as shown in the micro-CT image (Figure 1C,D), there are voids inside both the PEEK/SiN and PEEK TPMS scaffolds.

**Table 2:** Deviation of Porosity between Designed and Printed TPMS Scaffolds

material	designed porosity	printed porosity	deviation
PEEK	30%	24.75% ± 0.45%	–5.25%
	50%	43.45% ± 0.30%	–6.55%
	70%	69.15% ± 0.15%	–0.85%
PEEK/SiN	30%	29.68% ± 0.27%	–0.32%
	50%	47.77% ± 0.14%	–2.23%
	70%	71.16% ± 0.36%	1.16%

### 3.2 Quasi-Static Mechanical Testing Results

**Figure 2:**
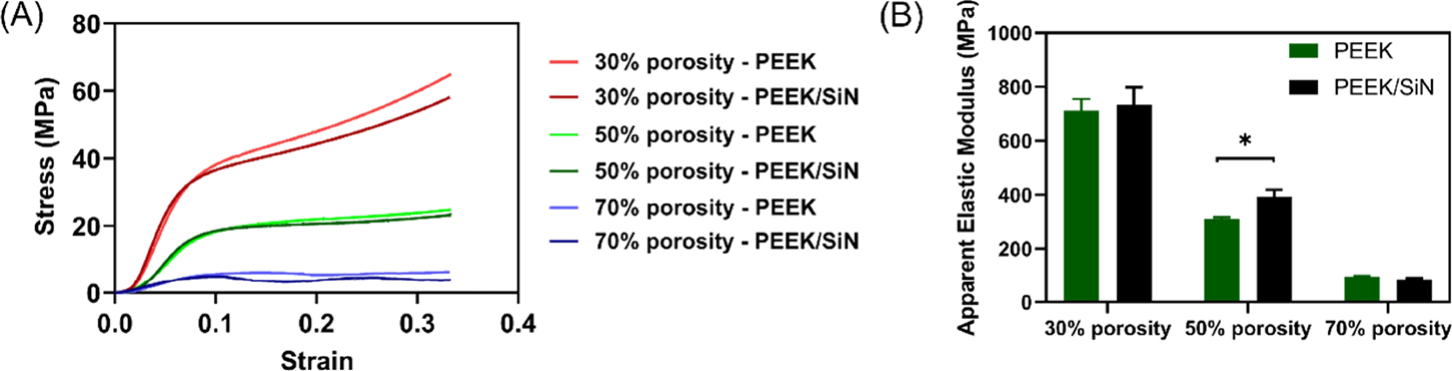
(A) Stress–strain curves and (B) Apparent elastic modulus of PEEK and PEEK/SiN scaffolds with different porosities under vertical compression.

Both PEEK and PEEK/SiN TPMS scaffolds were 3D-printed with three different porosities and tested under quasi-static conditions. The direction of loading was parallel to the build direction. As they underwent compression, the TPMS scaffolds with 50 and 70% porosity exhibited an initial linear elasticity and a subsequent long plateau stress stage. However, while scaffolds with 30% porosity experienced no plateau stress stage, they underwent a densification stage after the yield point. The apparent elastic modulus decreased with increasing porosity. As shown in Figure 2A, the stress–strain curves for the PEEK and PEEK/SiN scaffolds are similar. The deformation behavior of the gyroid TPMS scaffold is dominated by bending, which is typical of open cellular structures.^22^

**Figure 3:**
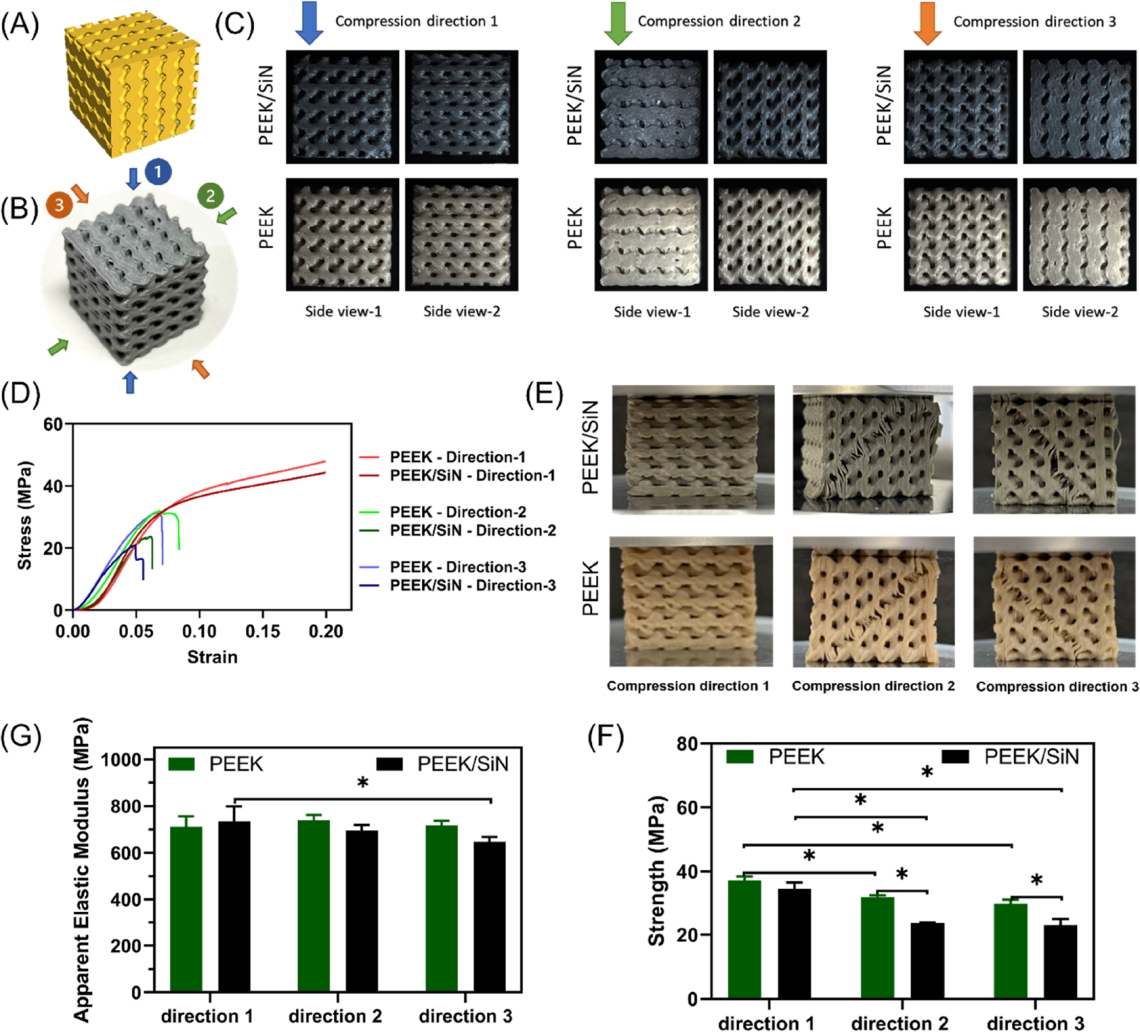
(A) Design model of Gyroid TPMS structure with a porosity of 30%; (B) 3D-printed PEEK/SiN gyroid TPMS scaffold with a porosity of 30% (arrows represent different compression directions); (C) side views of scaffolds under different compression direction; (D) stress–strain curves; (E) optical images of scaffolds after compression; (G, F) apparent elastic modulus and strength of PEEK and PEEK/SiN scaffolds under compression from different directions.

As can be seen in Figure 3D, the mechanical properties are higher in the parallel direction (compression direction 1) compared to the perpendicular direction (compression directions 2 and 3). In the parallel direction, the layers either slid over one another or structurally buckled when the stresses reached their maximum values, maintaining their structural integrity, even when high engineering strains of >30% were applied. However, in the perpendicular direction, delamination occurred between the layers as the compression load increased, leading to failure of the structure at relatively small strains. A 45° plane shear failure was observed as well. There was no significant difference in apparent elastic moduli between most groups (∼700 MPa), except between PEEK/SiN scaffolds with 30% porosity in compression directions 1 and 3. However, both PEEK and PEEK/SiN scaffolds showed yield strengths in the parallel direction higher than the ultimate strengths in the two perpendicular directions. Note that although the yield strengths of PEEK and PEEK/SiN scaffolds in the parallel direction were not significantly different (37.26 ± 1.27 and 34.56 ± 1.91 MPa, respectively), the PEEK scaffolds showed higher strength than the PEEK/SiN scaffold in both perpendicular directions.

**Figure 4:**
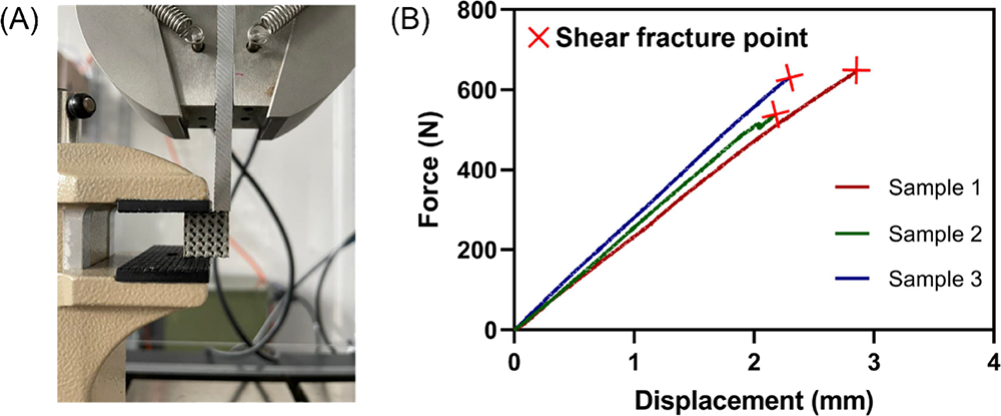
(A) Interfacial shear test setup on the Instron testing machine. Custom adaptors were attached to the clamps and the shear test was performed at 1 mm/min. (B) Force–displacement curve of PEEK/SiN TPMS scaffold.

To evaluate the interfacial connection layer by layer, we performed interfacial shear tests on PEEK/SiN TPMS scaffolds. The results showed that the interfacial shear strength was 8.09 ± 0.77 MPa for PEEK/SiN TPMS scaffolds with 30% porosity (Figure 4).

### 3.3 Progressive Loading Tests

**Figure 5:**
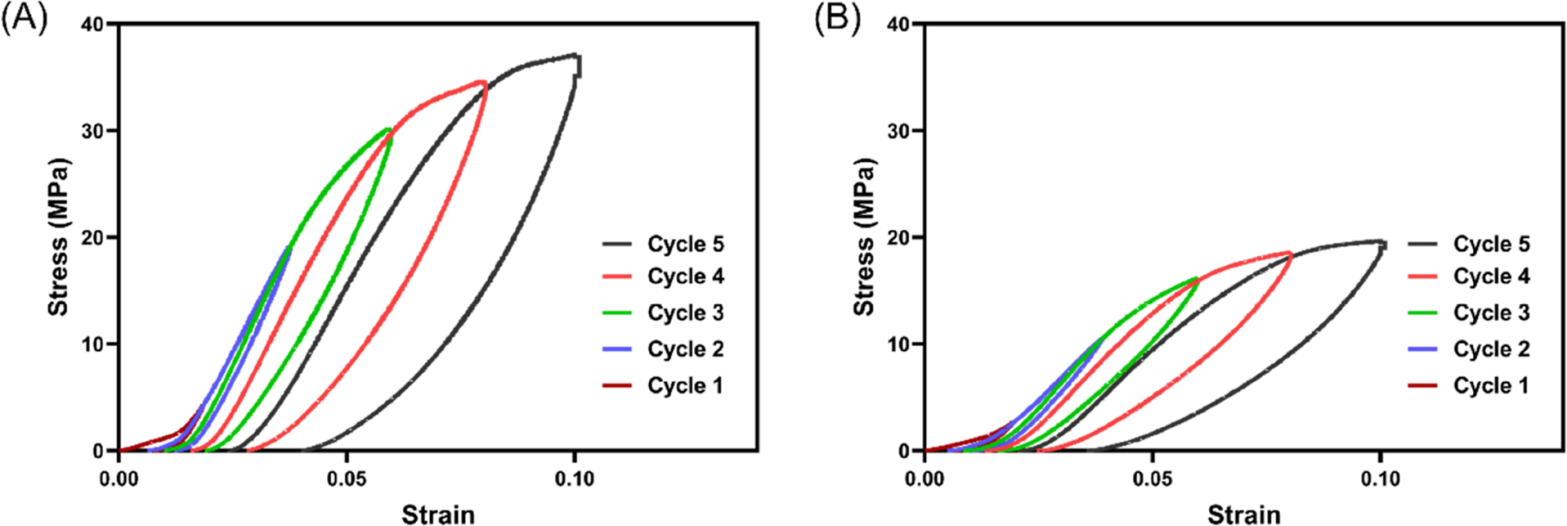
Stress–strain curves of PEEK/SiN TPMS scaffolds under progressive loading: (A) 30% porosity and (B) 50% porosity.

Since PEEK/SiN TPMS scaffolds with 30% and 50% porosity showed better mechanical properties and printability than the scaffold with 70% porosity, we applied dynamic loading tests on these two groups of scaffolds. Figure 5 shows the stress vs strain curve of the PEEK/SiN TPMS scaffold with 30 and 50% porosity under progressive loading. Due to the uneven surface, there is a toe region at the beginning of the loading. A large hysteresis loop was observed for both scaffolds with 30% porosity and 50% porosity, indicating good energy dissipation. In addition, the PEEK/SiN TPMS scaffold with 30% porosity showed higher strength than that of the scaffold with 50% porosity.

### 3.4 FEA Results

**Figure 6:**
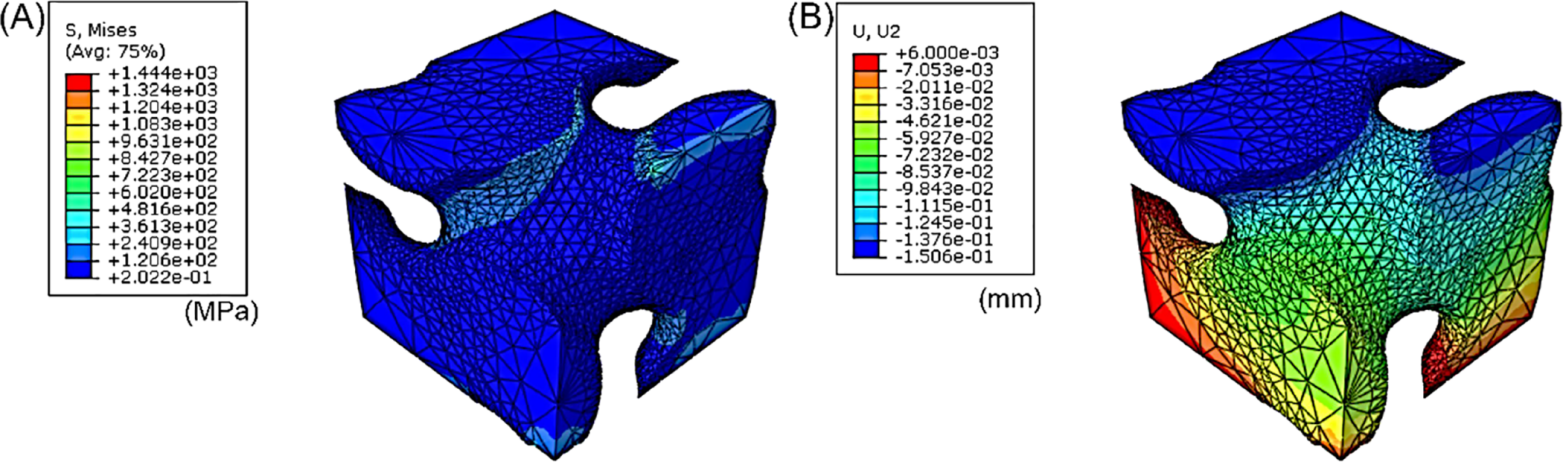
TPMS unit cell FE simulation: (A) stress; (B) displacement (final height after compression).

**Figure 7:**
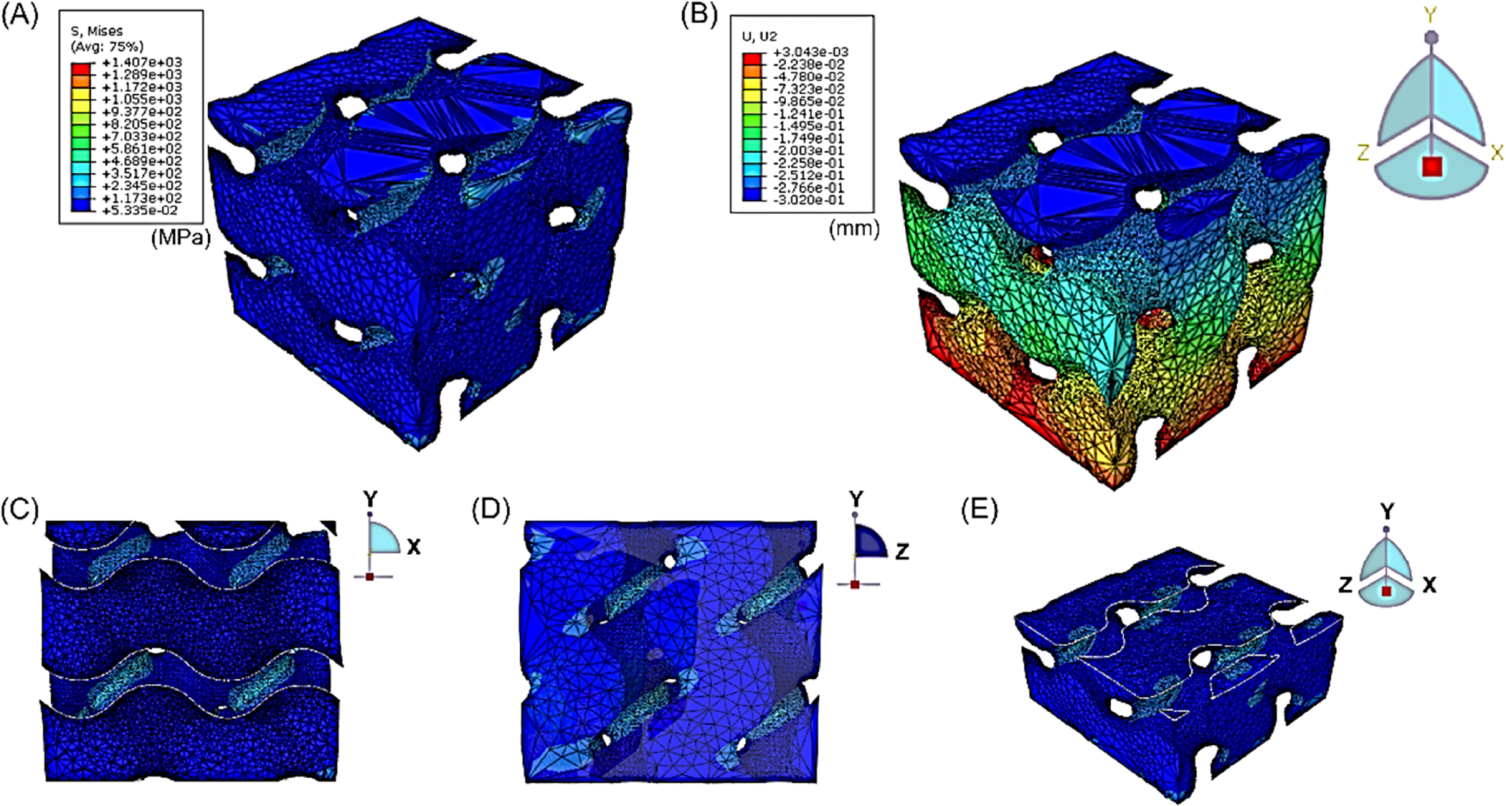
TPMS scaffold FE simulation: (A) stress; (B) displacement (final height after compression); (C) stress in the *Y*-*X* section; (D) stress in the *Y*-*Z* section; (E) stress in the cross-section.

Finite element analysis (FEA) was performed to investigate the mechanical properties of the TPMS scaffold and the unit cell under compressive loading. As shown in Figure 6, the results indicate that the neck of the unit cell experienced higher Von Mises stresses, up to 1444 MPa at a compression strain of 5%. Then, the TPMS scaffold with 2 × 2 × 2 cells was also examined to check the differences in mechanical behavior. As shown in Figure 7, a similar degree of stress distribution was observed compared to that of the unit cell. The simulated elastic modulus was 856 MPa for the TPMS scaffold with 2 × 2 × 2 cells and 886 MPa for the single unit cell. The simulated elastic modulus was slightly higher than the experimentally measured elastic modulus value (734 ± 64 MPa), which may be due to unavoidable defects in the printed scaffold.

### 3.5 In Vitro Cellular Responses of Preosteoblast Cells to the PEEK/SiN and PEEK TPMS Scaffolds

**Figure 8:**
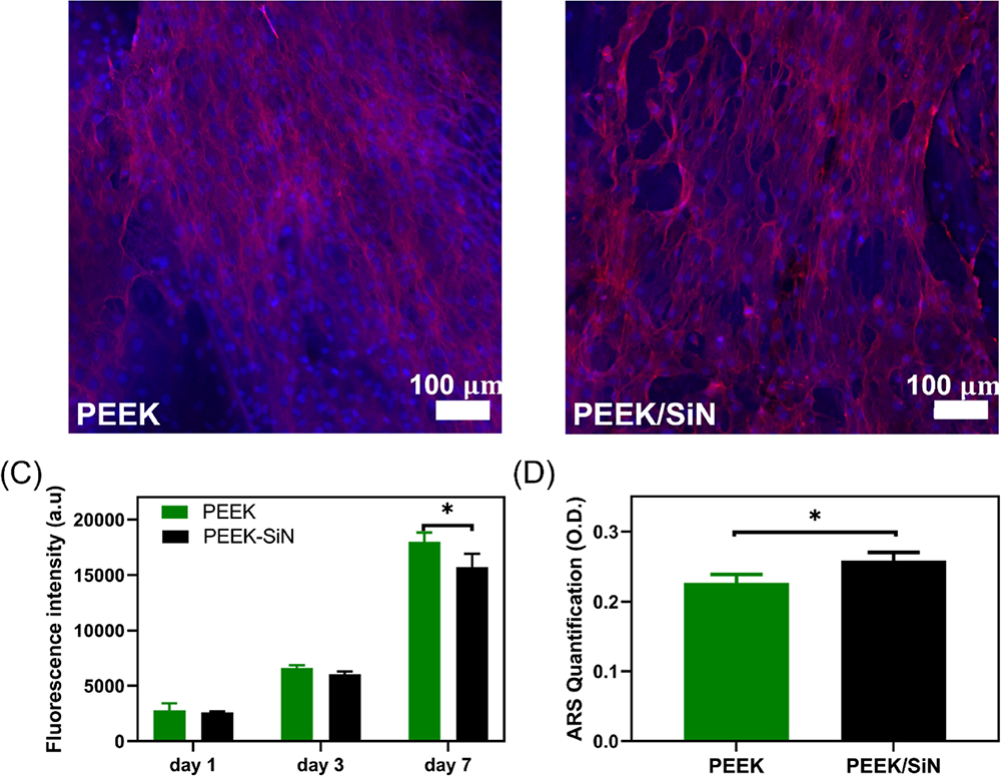
Representative CLSM images that show the staining of actin microfilament cytoskeletal protein (red) and nuclei counterstained with DAPI (blue) of the cells after 5 days of culturing on (A) PEEK scaffold and (B) PEEK/SiN scaffold; (C) PrestoBlue results of PEEK and PEEK/SiN scaffolds on days 1, 3, and 7 (significant difference appeared on days 7 with a P value of 0.0038); (D) quantitative analysis of mineralization by Alizarin red S (ARS) staining after 10 days (significant difference appeared on days 7 with a *P* value of 0.0286).

Mouse preosteoblast cells (MC3T3-E1) were used to investigate the cellular response of preosteoblast cells to the PEEK/SiN TPMS scaffolds. The PEEK/SiN TPMS scaffold with 30% porosity was chosen as the experimental group since it showed the best mechanical properties among the groups, matching as a minimum requirement the properties of cancellous bone, while a pure PEEK TPMS scaffold with 30% porosity was chosen as the control group. To evaluate the cellular morphology of cells cultured on the scaffolds, phalloidin and DAPI staining were performed. The cells exhibited strong attachment and spreading on both the PEEK and PEEK/SiN scaffolds (Figure 8). The proliferation of the cells was further confirmed by the results of the PrestoBlue assay.

**Figure 9:**
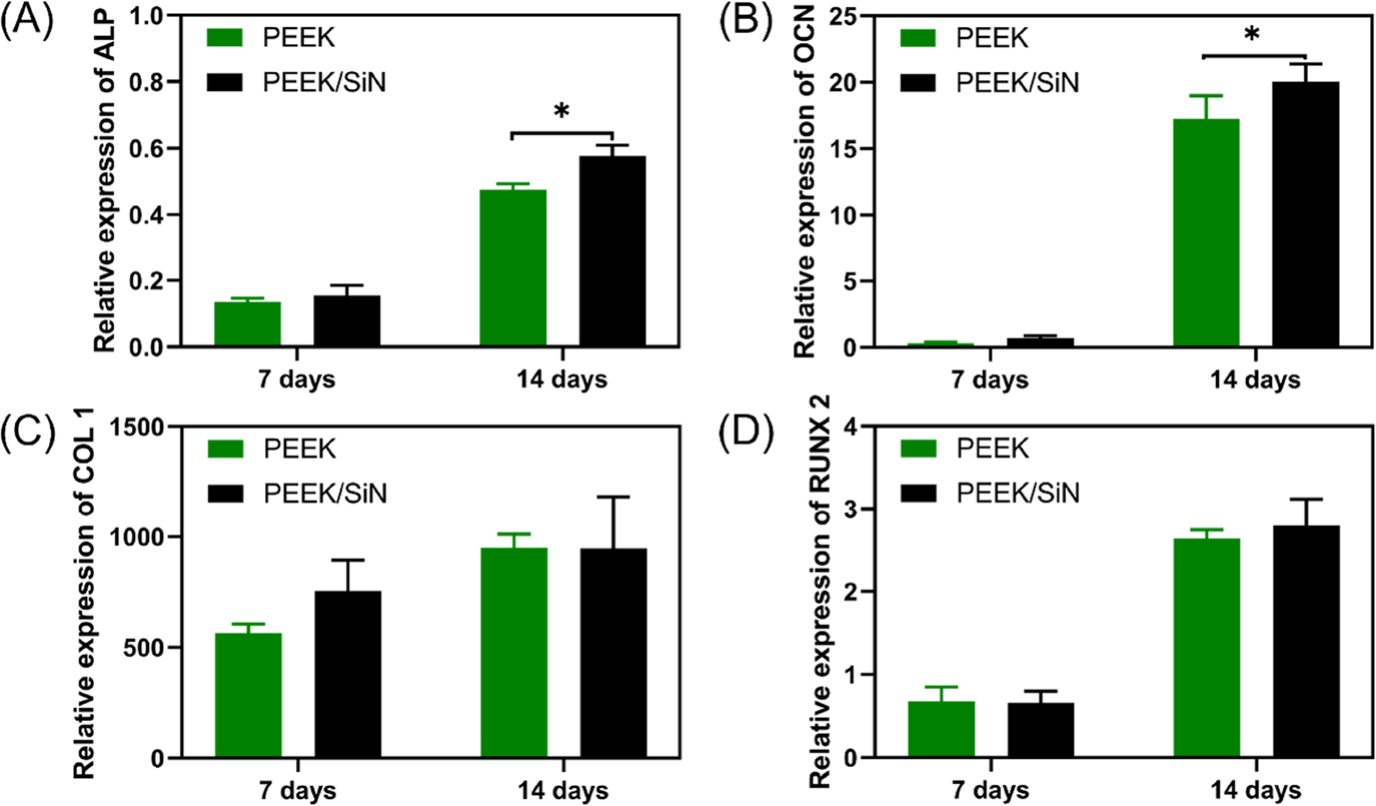
Osteogenic expression of (A) *ALP*, (B) *OCN*, (C) *COL 1,* and (D) *RUNX 2* for MC3T3-E1 cultured on PEEK and PEEK/SiN scaffolds by RT-qPCR analysis after 7 and 14 days. (Significant difference appeared on day 14 for ALP marker with a *P* value of 0.0017 and for the OCN marker with a *P* value of 0.0304).

The mineralization and osteogenic effect of the PEEK/SiN TPMS scaffolds were evaluated via ARS and gene expression tests. The PEEK/SiN scaffold showed significantly higher calcium deposition on day 10, exhibiting a higher mineralization. The differentiation of MC3T3-E1 cells on PEEK/SiN scaffolds was further assessed by measuring the expression of osteogenic markers such as ALP, OCN, RUNX2, and COL1 at 7 and 14 days. It was found that the osteogenic related gene expression (*ALP* and *OCN*) of MC3T3-E1 was upregulated on the PEEK/SiN scaffolds compared to the PEEK scaffolds after a 14-day culture, indicating that the silicon nitride addition to the PEEK scaffolds promotes osteogenic differentiation (Figure 9).

## 4 Discussion

Silicon nitride has been used for spinal fusion cages in the treatment or correction of intervertebral problems, such as spinal stenosis, spondylolisthesis, and disc herniation, for a number of years now. The flexural strength and elastic modulus of silicon nitride are estimated to be approximately 800–1100 MPa and 296–313 GPa, respectively.^28^ Although adequate strength is essential for implant safety, a high elastic modulus may lead to stress shielding and result in bone atrophy^29^ and even implant subsidence. This issue is particularly prevalent in commonly used spinal fusion cages. For instance, dense titanium exhibits an elastic modulus ranging from 55 to 114 GPa,^30^ while dense PEEK has an elastic modulus of 3.7 to 4.0 GPa.^23^ The design of porous structures presents a potential solution for decreasing the stiffness of implants; however, this often comes at the expense of strength.^31^ In our previous study,^14^ we investigated the correlation between the mechanical properties and porosity of silicon nitride bioceramics. Porous silicon nitride with a porosity of 70% exhibited a Young’s modulus of 14.84 ± 0.91 GPa, which is considerably higher than the structural modulus of human cancellous bone (ranging from a few hundred MPa to 2–3 GPa^32,33^). Moreover, porous silicon nitride remains brittle and lacks plasticity. In the present study, we found that the elastic moduli of PEEK/SiN TPMS scaffolds with 30% porosity fell within the same range as human cancellous bone. When the elastic modulus of an implant material closely matches that of human bone, it can help minimize the detrimental effects of stress shielding. By closely resembling the mechanical properties of the surrounding bone, the implant can distribute mechanical forces more uniformly, enabling the bone to bear its intended load. This characteristic is vital in maintaining the structural integrity of the bone and preventing excessive bone resorption or weakening.^34^ Additionally, the absence of stress shielding effects facilitates optimal stress distribution, which promotes bone cell attachment and growth around the implant, potentially enhancing osseointegration.^35^ The axial compressive strength of human vertebral bone was reported as 2.270 ± 1.142 MPa.^36^ The yield strength of the PEEK/SiN TPMS scaffold with 30% porosity was 34.56 ± 1.91 MPa. Two failure mechanisms were observed in the TPMS scaffolds under parallel compression: plastic yielding and local buckling. One advantage of TPMS structures over strut-based cellular structures is that the strut size varies linearly from one node to another, resulting in a gradual change in the material distribution. This eliminates the sudden increase/decrease in material distribution, which improves the mechanical properties.^22^ In addition, the unique geometry of the surface-based lattices reduces stress concentrations, thus producing a smoother crush behavior as they undergo compressive loading.^20^ Therefore, the PEEK/SiN TPMS scaffold limits the risk of implant failure and has demonstrated sufficient load-bearing capacity to function as a substitute for trabecular vertebral bone. Meanwhile, we performed FEA to obtain the elastic modulus and observe the internal stress distribution. The results showed that the simulated elastic modulus was in the same range as the experimental data, indicating that FEA has the potential to predict the elastic modulus of PEEK/SiN TPMS scaffolds over a wider range of porosities, which is helpful during the design process.

Although other additive manufacturing techniques, such as selective laser sintering (SLS) and stereolithography (SLA), can have better resolution than FDM when printing TPMS structures, the choice of materials is limited. For example, SLS is usually used to print metals while SLA requires resin as the base material.^37^ PEEK is a high-performance polymer known for its exceptional thermomechanical properties, making it highly desirable for diverse applications. However, the unique characteristics of PEEK present specific challenges in the context of 3D printing, requiring careful consideration of various factors to overcome these challenges. These factors encompass selecting the appropriate 3D printing parameters, ensuring the quality of the filament feedstock, addressing issues related to warping and bed adhesion, and implementing suitable postprocessing techniques.^38^ Currently, PEEK is available in the filament form for most fused deposition modeling/fused filament fabrication (FDM/FFF) machines, and there is a gradual emergence of PEEK in the powder form for SLS processes. Notably, EOS, a prominent manufacturer, has pioneered the use of selective laser sintering for printing PEEK.^39^ However, achieving high-temperature 3D printing with SLS presents challenges due to the potential leakage of extremely high temperatures beyond the model boundary, which can compromise the integrity of other powders in the build chamber. Furthermore, it should be noted that high-temperature materials tend to entail higher costs in the domain of 3D printing.^40^ In our study, we used the FDM printing technique, which is fast and cost-effective. However, it has the disadvantage that the resolution is diminished, and the connection between each layer may not be as strong, leading to anisotropic printed scaffolds. Furthermore, the actual infill density cannot ideally reach 100% with FDM. We also tested the mechanical properties of TPMS scaffolds perpendicular to the building direction. Although the ultimate strength decreased compared to the parallel direction, the value was still above 20 MPa, which is significantly higher than the compressive strength of human vertebrae. We also observed that the PEEK/SiN scaffold showed lower strength in both perpendicular directions than did the PEEK scaffold. Ceramic inclusions acting as flaws may diminish the connecting bonds between the layers, as they experience loading perpendicular to the build direction. The interfacial shear strength of the PEEK/SiN scaffold between the printed layers was determined by a shear test. The shear strength of trabecular bone is generally much weaker than the compressive strength,^41^ usually with a value of less than 8 MPa.^42,43^ Therefore, the PEEK/SiN TPMS scaffold with 30% porosity is also strong enough to withstand shear force as a substitute for trabecular bone.

As natural spinal tissues have unique dynamic properties, it is essential to consider damping properties, when designing spinal implants. A direct method that measures energy dissipation during dynamic progressive loading was applied on PEEK/SiN scaffolds with 30 and 50% porosity. No catastrophic fracture of the TPMS scaffolds was observed during compression. Instead, a pronounced recovery was seen upon unloading. A large hysteresis loop was observed for both, substantially larger than that of the porous silicon nitride that we measured in our previous study.^14^ Generally, energy dissipation can be attributed to intrinsic material damping, elastic buckling, or plastic deformation, with both the material composition and structure playing a role in energy dissipation. In our study, the PEEK/SiN scaffold retains the intrinsic material-damping properties of PEEK. Meanwhile, the TPMS structure also leads to high energy dissipation due to its periodic cells and interconnected open cavities.

Regarding the interaction between cells and material, the attachment and osteogenic differentiation of MC3T3-E1 cultured on the PEEK and PEEK/SiN scaffolds were investigated. Both scaffolds demonstrated excellent biocompatibility. ARS is an effective measure of calcium deposition and thus detects osteogenic induction. The expression of genes related to osteogenesis, such as *OCN, ALP, RUNX2*, and *COL1*, act as important osteogenic markers in the process of bone regeneration. Consistent with ARS analysis, PEEK/SiN scaffolds showed a significant enhancement in the expression of *ALP* and *OCN*. *ALP* is an enzyme that is synthesized by active osteoblasts and plays a critical role in initiating the mineralization of newly formed bone tissue. *OCN* is a noncollagenous protein present in the extracellular bone matrix that facilitates bone formation and has a strong affinity for calcium ions during the mineralization process.^44^ Increased expression of ALP and OCN is a significant marker of osteogenic differentiation, which refers to the transformation of undifferentiated mesenchymal stem cells into mature osteoblasts responsible for bone formation. These findings suggest that the PEEK/SiN scaffold plays a crucial role in the mineralization process. Moreover, these results align with previous studies highlighting the superior properties of silicon nitride in stimulating osteoblast differentiation and promoting new bone formation.^11,45−47,48^ For example, Lee et al.^45^ developed a silicon nitride reinforced gelatin/chitosan cryogel system (SiN-GC) by incorporating silicon nitride microparticles into a gelatin/chitosan cryogel (GC). Their findings confirmed enhanced cell proliferation, mineralization, and upregulation of osteogenic genes in MC3T3-E1 preosteoblast cells cultured on SiN-GC scaffolds compared to GC scaffolds. These observations were observed under both static cell culture conditions and simulated physiological conditions by subjecting the scaffolds to cyclic compressive loading in a bioreactor.

Determining the clear mechanism behind the osteogenic behavior of silicon nitride is still a daunting challenge. However, many researchers have studied possible reasons for silicon nitride accelerating bone repair and inducing osseointegration. We have summarized them in the recently published review article of silicon nitride.^49^ Intuitively, silicon nitride is composed of two primary elements: silicon and nitrogen. When this material is immersed in an aqueous solution, Si–N bonding undergoes covalent cleavage. This leads to the spontaneous release of ammonia and the formation of a hydrated layer of silicon dioxide on the surface, which interacts synergistically with the human body (as depicted by eqs 3 and 4).^50^
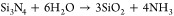
3
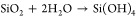
4

In one aspect, the presence of silanol groups (SiOH) can facilitate the mineralization of the interface, and the release of silicic acid can prevent the resorption of bone by osteoclasts by antagonizing the activation of signal transducers.^51^ In another aspect, surfaces modified with ammonia promote the activity of osteoblasts by enabling covalent coupling of proteins.^52^ Additionally, Y^3+^ is a key factor leading to the rapid folding of osteocalcin onto the surface of silicon nitride with a Y_2_O_3_ sintering additive.^47^ This osteogenic potential is further enhanced by the demonstrated antibacterial properties of silicon nitride, creating favorable conditions for bone deposition by inhibiting biofilm formation on an implant surface.^53^

In our study, we incorporated 10 wt % silicon nitride powder into the PEEK matrix, which is below the estimated percolation threshold of 30 wt % for silicon nitride. This choice was made to ensure that the modulus of the PEEK/SiN composite remains comparable to that of pure PEEK. However, increasing the silicon nitride content is likely to enhance the osteogenic properties of the composite scaffolds. Therefore, it is desirable to increase the silicon nitride content in the PEEK matrix, approaching but remaining below the percolation threshold, to achieve improved properties. The main challenge lies in effectively dispersing higher concentrations of silicon nitride powders within the PEEK matrix. Therefore, additional efforts should be dedicated to enhancing the compounding and extrusion processes of the PEEK/SiN filaments. It is also important to investigate the effect of the silicon nitride content on the mechanical and osteogenic properties of the composite scaffolds. Furthermore, conducting fatigue tests and in vivo studies are crucial steps for further research and exploration of this topic.

## 5 Conclusions

In this study, we aimed to fabricate PEEK/SiN TPMS scaffolds using FDM technology, which have a TPMS structure that offers numerous advantages, such as a large surface area and uniform stress distribution under load. The mechanical properties and dynamic behavior of the PEEK/SiN scaffolds with varying porosities were evaluated by mechanical testing and finite element analysis. The PEEK/SiN TPMS scaffold with 30% porosity exhibited an elastic modulus of 734 ± 64 MPa and a compressive strength of 34.56 ± 1.91 MPa, which are similar to those of trabecular bone. Furthermore, the PEEK/SiN scaffold demonstrated excellent damping properties in dynamic loading tests. In vitro studies were conducted to investigate the biological properties of the PEEK/SiN scaffolds using a PEEK scaffold as a control. The findings indicated that the PEEK/SiN scaffold promoted the proliferation and differentiation of mouse preosteoblast cells (MC3T3-E1) and resulted in improved expression of relevant osteogenic genes. In conclusion, the mechanical and biological tests demonstrated that PEEK/SiN TPMS scaffolds hold promise for use as spinal fusion implants and in bone tissue engineering applications. In the future, additional efforts should focus on increasing the silicon nitride content in PEEK to achieve better osteogenic response while maintaining the mechanical properties of PEEK.

## Data Availability

The raw/processed data required to reproduce these findings are available to download from https://data.mendeley.com/datasets/wgyrgt7n5j.
